# Prognostic significance of CT-determined sarcopenia in patients with advanced gastric cancer

**DOI:** 10.1371/journal.pone.0202700

**Published:** 2018-08-20

**Authors:** Jong Soo Lee, Young Saing Kim, Eun Young Kim, Wook Jin

**Affiliations:** 1 School of Medicine, Gachon University School of Medicine, Incheon, South Korea; 2 Division of Medical Oncology, Department of Internal Medicine, Gachon University Gil Medical Center, Incheon, South Korea; 3 Department of Radiology, Gachon University Gil Medical Center, Incheon, South Korea; 4 Laboratory of Molecular Disease and Cell Regulation, Department of Biochemistry, Gachon University School of Medicine, Incheon, South Korea; Universidade de Mogi das Cruzes, BRAZIL

## Abstract

**Background:**

Sarcopenia, defined as decreased skeletal muscle mass, is prevalent and associated with poor prognosis in various solid tumors. This study aimed to determine the prognostic role of sarcopenia in patients with advanced gastric cancer (AGC).

**Methods:**

This retrospective study consisted of 140 consecutive patients who underwent palliative chemotherapy for AGC. A cross-sectional area of muscle at the level of the third lumbar vertebra (L3) was measured using baseline computed tomography (CT) scans. Sarcopenia was defined as a L3 skeletal muscle index of ≤ 49 cm^2^/m^2^ for men and ≤ 31 cm^2^/m^2^ for women using Korean-specific cutoffs. We compared the overall survival (OS) and clinical characteristics of patients with and without sarcopenia.

**Results:**

The median age was 67 years, and 133 (95%) patients had metastatic disease. Sarcopenia was present in 67 patients (47.9%) and was significantly related to male sex (p < 0.001) and low body mass index (p = 0.002). Patients with sarcopenia had a significantly shorter OS than those without sarcopenia (median, 6.8 months vs. 10.3 months, respectively; p = 0.033). In the multivariable analysis, sarcopenia was an independent prognostic factor of poor OS (hazard ratio, 1.51, p = 0.029); no response to chemotherapy (p < 0.001), no second-line chemotherapy (p < 0.001), metastatic sites ≥ 3 (p < 0.001), and low serum albumin level (p = 0.033) were also independent prognostic factors of poor OS.

**Conclusion:**

Sarcopenia, as determined by baseline CT, can be used to predict poor prognosis in AGC patients treated with palliative chemotherapy.

## Introduction

Stomach cancer is the second most common cancer and the third leading cause of cancer-related death in Korea [1]. Early stomach cancer can be cured by surgical resection [2]. Although early detection is often possible with screening, more than half of all patients are diagnosed with inoperable advanced or metastatic disease requiring palliative chemotherapy [3]. Weight loss, which is a key feature of cancer cachexia syndrome [4], is a common presentation in patients with advanced gastric cancer (AGC). However, weight loss may not always be accurately described by patients during medical examinations. In addition, body weight changes do not precisely reflect body composition changes. Moreover, weight loss is uncertain in patients with a large tumor mass or fluid collection, such as ascites or body edema.

Sarcopenia, defined by a loss of skeletal muscle mass, is an important age-related health issue in elderly people. It is associated with physical disability, injury, and mortality. Recently, the clinical significance of sarcopenia in patients with various forms of cancer is being increasingly recognized; sarcopenia is an independent prognostic factor associated with a decreased survival and an increased risk of chemotherapy toxicity in various forms of cancer, including small cell lung cancer, breast cancer, hepatocellular carcinoma, and urothelial cancer [5–10].

Although a previous Japanese cohort study revealed low skeletal muscle mass is not associated with poor prognosis in patients with metastatic gastric cancer receiving chemotherapy, the study used cut points derived from a Caucasian population [11]. The primary objective of this study was to evaluate the prognostic role of computed tomography (CT)-determined skeletal muscle index using ethnic specific cut-offs for sarcopenia in AGC patients receiving palliative chemotherapy.

## Patients and methods

### Patients

The medical records of consecutive patients diagnosed with AGC between January 2012 and December 2015 were screened using a prospectively maintained gastric cancer database. Among these patients, the selection criteria for analysis were as follows: patients who had histologically proven gastric cancer, underwent first-line palliative chemotherapy, and had adequate quality abdominopelvic (AP) CT scans within 4 weeks of initiation of first-line chemotherapy.

Clinicopathological data included age, sex, height, weight, Eastern Cooperative Oncology Group performance status (ECOG PS), human epidermal growth factor receptor 2 (HER2) status, baseline laboratory values (complete blood count with differential count and serum chemistry), disease status (locally advanced vs. metastatic), metastatic site, previous treatment history, chemotherapy regimen, chemotherapy response, survival status, and dates of administration of chemotherapy and the last follow-up. Response evaluation was performed according to the Response Evaluation Criteria in Solid Tumors version 1.1 using follow-up radiographic images obtained every 6 to 8 weeks during treatment.

Body mass index (BMI) was calculated as weight divided by height squared (kg/m^2^), and BMI values were categorized as underweight (< 18.5 kg/m^2^), normal (18.5−22.9 kg/m^2^), overweight (23.0−24.9 kg/m^2^), or obese (≥ 25 kg/m^2^) [12].

Ethics approval for this study was granted by the Gil Medical Center Institutional Review Board (approval number: GBIRB-2017-218).

### Image analysis

CT images from AP CT scans were retrospectively analyzed by a radiologist (EYK) who is blinded to clinical outcome of the patients. The third lumbar vertebra (L3) was selected as the landmark since the cross-sectional area of skeletal muscle mass in this region was found to be most correlated with total body SMM (*r* = 0.924, *p* < 0.001) in the general population [13–15].

To quantify skeletal muscle mass using AP CT, commercially available software (Terarecon version 3.4.2.11, San Mateo, CA) was used. After applying threshold methods using a predefined Hounsfield unit (HU) threshold of -25 to 150 HU for skeletal muscle mass, muscle boundaries were corrected manually when necessary to obtain cross-sectional areas (cm^2^) for skeletal muscle. L3 skeletal muscle index (SMI, cm^2^/m^2^) was defined as the cross-sectional area of muscle at the L3 level normalized for stature as is conventional for BMI.

### Definition of sarcopenia

Sarcopenia was defined as a L3 SMI of ≤ 49 cm^2^/m^2^ for men and ≤ 31 cm^2^/m^2^ for women using cutoff points specific for the Korean population, based on the Korea National Health and Nutrition Examination Study (KNHANES). A previous epidemiologic study evaluated height-adjusted appendicular skeletal muscle mass (ASM; kg/m^2^) of a reference group of young Koreans using dual-energy X-ray absorptiometry (DXA) and determined sarcopenia cutoff values of 6.58 and 4.59 kg/m^2^ for Korean men and women, respectively [16]. It was reported that L3 muscle area by CT and ASM by DXA are linearly related; L3 SMI cutoff values were calculated using the regression equation [13]:

L3 SMI (cm^2^/m^2^) = [height-adjusted ASM (kg/m^2^) - 1.17] / 0.11.

### Statistical analysis

Descriptive statistics were reported as proportions or medians with ranges. Comparisons between subjects with and without sarcopenia were performed using Pearson’s χ^2^ test or Fisher’s exact test for categorical variables. Continuous variables were analyzed using Student’s t-test or the Mann–Whitney U test. Survivals were estimated using the Kaplan–Meier method and compared using the log-rank test. Overall survival (OS) was defined as the time from the date of initiation of first-line chemotherapy to the date of death or last follow-up. Multivariable Cox proportional hazard models were used to identify prognostic factors for survival. Variables with a p-value of < 0.05 by the log-rank test were included in the multivariable analysis, and backward regression was used. Two-sided p-values < 0.05 were considered statistically significant. The analysis was performed using the open-source statistical software R version 3.3.1 (R Foundation, Vienna, Austria, http://www.r-project.org).

## Results

### Characteristics of the study population

A total of 140 consecutive patients were included in this study (Table 1). Among them, 106 patients (75.7%) were male, and 133 (95.0%) had metastatic disease. Forty-eight patients (34.3%) were HER2-positive. The average BMI was 21.4 ± 3.3 kg/m^2^ and 22.9% of the patients were underweight (n = 32). The mean L3 SMI was 46.6 ± 9.1 cm^2^/m^2^ (range: 16.1−72.6 cm^2^/m^2^) for men and 38.7 ± 5.1 cm^2^/m^2^ (range: 30.5−49.5 cm^2^/m^2^) for women.

**Table 1:** Patient characteristics according to the presence of sarcopenia.

Characteristics	Sarcopenia (n = 67)	No sarcopenia (n = 73)	p value
**Age (years)**			
Median (range)	69 (29–83)	66 (22–91)	0.061
≥ 70 years	32 (47.8%)	25 (34.2%)	0.104
**Male**	66 (98.5%)	40 (54.8%)	<0.001
**Extent of disease**			0.786
Locally advanced	3 (4.5%)	4 (5.5%)	
Metastatic*	64 (95.5%)	69 (94.5%)	
**ECOG performance status**			0.271
0–1	51 (76.1%)	60 (83.6%)	
≥ 2	16 (23.9%)	12 (16.4%)	
**HER2(+)**	22 (32.8%)	26 (35.6%)	0.729
**First-line regimen**			0.968
S-1/cisplatin or XP	20 (29.9%)	22 (30.1%)	
FOLFOX or XELOX	17 (25.4%)	16 (21.9%)	
Trastuzumab plus XP	12 (17.9%)	16 (21.9%)	
FOLFIRI	3 (4.5%)	5 (6.8%)	
Modified DCF	3 (4.5%)	2 (2.7%)	
Trastuzumab plus capecitabine	9 (13.4%)	10 (13.7%)	
S-1 or capecitabine	3 (4.5%)	2 (2.7%)	
**Response to first-line therapy**			0.363
Responder	27 (40.3%)	35 (47.9%)	
Non-responder	40 (59.7%)	38 (52.1%)	
**Receipt of second-line therapy**	23 (34.3%)	35 (47.9%)	0.102
**Second-line regimen**^†^			0.454
Pacltiaxel	9 (39.1%)	15 (42.9%)	
Irinotecan	6 (26.1%)	7 (20.0%)	
FOLFIRI	2 (8.7%)	8 (22.9%)	
FOLFOX	2 (8.7%)	3 (8.6%)	
Ramucirumab plus paclitaxel	2 (8.7%)	2 (5.7%)	
Docetaxel	2 (8.7%)	0	
**Number of metastatic sites**			0.576
0–2	54 (80.6%)	56 (76.7%)	
≥ 3	13 (19.4%)	17 (23.3%)	
**BMI**			0.002
Underweight	23 (34.3%)	9 (12.3%)	
Normal	29 (43.3%)	31 (42.5%)	
Overweight	12 (17.9%)	18 (24.7%)	
Obese	3 (4.5%)	15 (20.5%)	
**L3 SMI (cm**^**2**^**/m**^**2**^**),** median (range)	42.4 (16.1–48.7)	49.8 (31.9–72.6)	<0.001
**Hemoglobin (g/dL),** median (range)	10.2 (7.2–15.0)	11.2 (7.2–15.8)	0.082
**Serum albumin (g/dL)**			
median (range)	3.4 (2.4–4.5)	3.6 (1.9–4.5)	0.114
< 3.5 g/dL	33 (49.3%)	24 (32.9%)	0.072
**Neutrophil-lymphocyte ratio,** median (range)	3.8 (1.1–45.6)	3.0 (1.0–22.3)	0.085
**CRP (g/dL),** median (range)	2.97 (0.02–20.17)	1.45 (0.01–18.04)	0.069

*BMI*, body mass index; *CRP* C-reactive protein; *DCF*, docetaxel, cisplatin, and 5-fluorouracil; *ECOG*, Eastern Cooperative Oncology Group; *FOLFIRI*, 5-fluorouracil, leucovorin, and irinotecan; *FOLFOX*, 5-fluorouracil, leucovorin, and oxaliplatin; *HER2*, human epidermal growth factor 2; *SMI*, skeletal muscle index; *XELOX*, capecitabine and oxaliplatin *XP*, capecitabine and cisplatin.

*Four patients in the sarcopenia group and three in the non-sarcopenia group had recurrent disease.

^†^The percentage was calculated only within patients treated with the second-line treatment.

### Prevalence of and factors associated with sarcopenia

The overall prevalence of sarcopenia was 47.9% (62.3% for men and 2.9% for women). In elderly patients (aged ≥ 70 years), the overall prevalence of sarcopenia was 56.1% (90.0% for men and 0% for women). The clinical characteristics of patients with and without sarcopenia are summarized in Table 1. The prevalence of sarcopenia was related to male sex (p < 0.001) and low BMI (p = 0.002). No significant difference was found between patients with and without sarcopenia regarding age, the extent of the disease, ECOG PS, HER2 status, first-line regimen, response to chemotherapy, receipt of second-line therapy, number of metastatic sites, hemoglobin level, serum albumin level, neutrophil to lymphocyte ratio, and C-reactive protein level.

### Prognostic significance of sarcopenia in AGC patients

**Fig 1:**
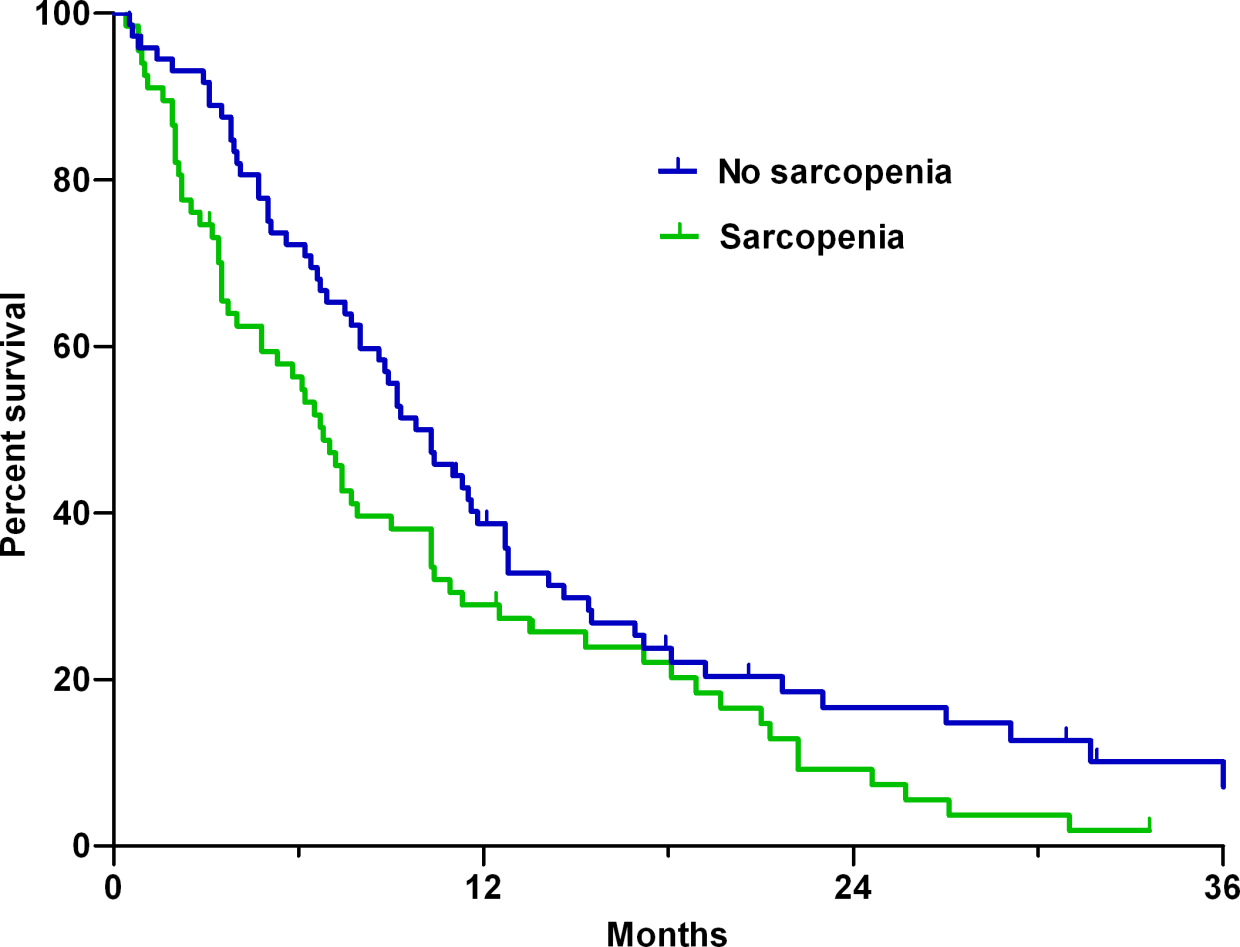
Kaplan–Meier estimates of overall survival in patients with and without sarcopenia.

Over a median follow-up of 31.9 months (95% confidence interval [CI]: 28.8–34.9 months), 124 patients (88.6%) died. For all patients, the median OS was 8.6 months (95% CI, 6.7−10.4 months). Patients with sarcopenia had a significantly shorter median OS than those without sarcopenia (6.8 vs. 10.3 months, respectively; p = 0.033 by the log-rank test; Fig 1).

Univariable analysis showed that in addition to the presence of sarcopenia, ECOG PS ≥ 2, no response to chemotherapy, no second-line chemotherapy, low serum albumin level (< 3.5 g/dL), and metastatic sites ≥ 3 were significant prognostic factors (Table 2). Multivariable analysis showed that sarcopenia was an independent prognostic factor for a shorter OS (hazard ratio [HR], 1.51; 95% CI, 1.04−2.18; p = 0.029), along with no response to chemotherapy (HR, 2.47; 95% CI, 1.69−3.61, p < 0.001), no second-line chemotherapy (HR, 2.43; 95% CI, 1.64−3.59, p < 0.001), metastatic sites ≥ 3 (HR, 2.31; 95% CI, 1.49−3.60; p < 0.001), and low serum albumin level (HR, 1.50; 95% CI, 1.03−2.19; p = 0.033).

**Table 2:** Results of univariable and multivariable analyses of overall survival.

Variables	Univariable analysis		Multivariable analysis	
	HR (95% CI)	p value	HR (95% CI)	p value
Sarcopenia	1.47 (1.03–2.10)	0.033	1.51 (1.04–2.18)	0.028
Age ≥ 70	1.52 (1.06–2.17)	0.057		
Male sex	1.11 (0.73–1.67)	0.632		
ECOG PS ≥ 2	2.18 (1.42–3.37)	<0.001		
HER2(-)	1.25 (0.86–1.82)	0.238		
No response to chemotherapy	2.23 (1.55–3.22)	<0.001	2.47 (1.69–3.61)	<0.001
No 2^nd^-line therapy	1.75 (1.22–2.51)	0.002	2.43 (1.64–3.59)	<0.001
Underweight (BMI < 18.5)	1.40 (0.93–2.12)	0.105		
Metastatic sites ≥ 3	1.74 (1.14–2.64)	0.010	2.31 (1.49–3.60)	<0.001
Albumin < 3.5 g/dL	1.56 (1.09–2.23)	0.016	1.50 (1.03–2.19)	0.033

*BMI*, body mass index; *CI*, confidence interval; *ECOG PS*, Eastern Cooperative Oncology Group Performance Status; *HER2*, human epidermal growth factor 2; *HR*, hazard ratio

## Discussion

Sarcopenia, characterized by progressive and generalized loss of skeletal muscle mass [17], has been established as a key diagnostic criterion for cancer cachexia [4]. CT is regarded as the gold-standard method to evaluate body composition and the presence of sarcopenia [18]; it is also a useful tool for staging and response assessment of gastric cancer. Using baseline CT scans before palliative chemotherapy, we showed that sarcopenia is commonly present in patients with AGC (47.9%). Moreover, sarcopenia was identified as an independent prognostic factor for a poor OS.

The significance of sarcopenia in the management of gastric cancer has been analyzed mainly in patients who underwent gastrectomy. In previous studies including more than 100 patients individually, the prevalence of preoperative sarcopenia ranged from 12.5% to 57.7% [19–25]. Sarcopenia negatively affects the surgical treatment of stomach cancer patients. It is an independent risk factor for postoperative complications in patients undergoing radical gastrectomy [19, 22, 23, 25, 26] and patients with sarcopenia had a significantly shorter OS after surgery [20, 22, 27, 28]. Moreover, sarcopenia was associated with chemotherapy toxicity in gastric cancer patients in the perioperative setting, leading to early discontinuation of chemotherapy and dose reduction [29, 30]. At palliative setting of gastric cancer, a Japanese cohort study revealed low skeletal muscle mass is not associated with poor prognosis [11]. However, the sample size was small (n = 53) and they used skeletal muscle index cut points derived from a Caucasian population to determine sarcopenia, which would be inappropriate for Asian population. In the study, low skeletal muscle density caused by increase in lipid content of muscle was an independent predictor of poor outcome, which would indicate the muscle quality change precede the quantity change.

The diagnostic criteria of sarcopenia remain controversial and cutoff values, used for defining sarcopenia, differed between studies. The cutoffs for L3 SMI to define sarcopenia in gastric cancer ranged from 36.0 to 53.0 cm^2^/m^2^ in men and from 29.0 to 41.0 cm^2^/m^2^ in women [19–25]. The European Working Group on Sarcopenia in Older People recommends the use of data from healthy young adults, with cutoff points at two standard deviations below the mean reference value [17]. Therefore, the definition of sarcopenia can vary depending on the characteristics of the reference population, such as age, race, and country. The SMI cut-off as recommended by cancer cachexia consensus is 55.4 cm^2^/m^2^ for men and 38.9 cm^2^/m^2^ for women, which is based on data from 229 non-Hispanic white men and women aged 18 to 40 years who were participants in the Rosetta Study (1986–1992) [31, 32]. In contrast, we used Korean-specific diagnostic criteria for sarcopenia: individuals aged 20 to 29 years (1,047 men and 1,433 women) who participated in KNHANES (2008–2010) were used as the young reference group to determine the sarcopenia cutoff points [33]. More research is needed to obtain good reference values for various ethnic groups and cancer patients.

In this study, sarcopenia appears to be dramatically more prevalent in men compared with women (62.3% vs. 2.9%). This would reflect ethnic characteristics of the extremely low prevalence of sarcopenia in Korean women. A recent epidemiologic study reported extremely low prevalence of sarcopenia in Korean women (prevalence of sarcopenia, 12.4% vs 0.1% for elderly men and women in Korean population of ≥ 65 years) [16], in comparison with similar prevalence of sarcopenia between sex in Western countries (prevalence of sarcopenia for elderly men and women, 25.7% and 23.1% in Germany [34], 26.8% and 22.6% in America [35]).

There are several potential explanations for the relationship between sarcopenia and poor prognosis in patients with advanced cancer. First, tumors with a more aggressive behavior tend to have a higher metabolic activity that may lead to sarcopenia [36]. However, we found no difference in the response to chemotherapy between patients with and without sarcopenia (p = 0.583). Second, several studies have suggested that myokines, hormones produced by skeletal muscle, may have anticancer effects [37, 38]. Therefore, the decrease in myokine secretion due to muscle loss may be related to cancer progression. Third, poor tolerance to chemotherapy can explain the negative effects of sarcopenia on survival [29, 30].

Previous study revealed muscle loss of more than 9% during chemotherapy was also independently associated with a shorter survival (HR, 4.47; 95% CI, 2.21−9.05; p < 0.001) [39]. This finding suggests that interventions to prevent sarcopenia or to increase muscle mass may be a potential therapeutic strategy to improve treatment outcomes in cancer patients. Physical exercise, specifically resistance training, can increase muscle mass and strength in people with age-related sarcopenia [40, 41]. Recent studies have evaluated exercise interventions (aerobic or resistance exercise, or both) in advanced cancer patients, and have suggested that supervised exercise interventions are safe and feasible [42]. In addition, it was reported that exercise interventions can maintain or improve muscle strength and physical function and may improve quality of life in advanced cancer patients [43]. Future research should aim to implement large, long-term, randomized trials to investigate the effects of exercise on mortality in advanced cancer. Studies on the effects of a combination of exercise and pharmacologic agents should also be performed. Currently, a phase III trial of multimodal intervention (exercise, nutrition, anti-inflammatory medication) is being conducted across a number of international sites (NCT02330926) [44].

The present study has several limitations that require consideration. First, the patients' number was small because this study was performed at a single institution. Second, we failed to get information regarding muscle function and body weight changes as a retrospective study design. Third, we did not evaluate the skeletal muscle density, although several studies have suggested that the skeletal muscle density may be superior to muscle mass in predicting survival [11, 45–47]. Further study is needed to evaluate the usefulness of these imaging biomarkers in detecting and monitoring sarcopenia.

In summary, sarcopenia, as determined by baseline CT, can be used to predict prognosis in patients with AGC who are under treatment with palliative chemotherapy. The identification of sarcopenia in AGC patients may enable early intervention to maintain and improve muscle mass. Further research is required to determine whether various therapies, including exercise for preventing sarcopenia, can improve the prognosis of cancer patients.
